# Psychogenic Aging: A Novel Prospect to Integrate Psychobiological Hallmarks of Aging

**DOI:** 10.1038/s41398-024-02919-7

**Published:** 2024-05-30

**Authors:** Manuel Faria, Ariel Ganz, Fedor Galkin, Alex Zhavoronkov, Michael Snyder

**Affiliations:** 1 Department of Genetics, Stanford University School of Medicine, Stanford, CA USA; 2 Department of Psychology, Stanford University, Stanford, CA USA; 3 Deep Longevity, Hong Kong, China; 4 Insilico Medicine, Hong Kong, China; 5 Buck Institute for Research on Aging, Novato, CA USA

**Keywords:** Predictive markers, Human behaviour

## Abstract

Psychological factors are amongst the most robust predictors of healthspan and longevity, yet are rarely incorporated into scientific and medical frameworks of aging. The prospect of characterizing and integrating the psychological influences of aging is therefore an unmet step for the advancement of geroscience. Psychogenic Aging research is an emerging branch of biogerontology that aims to address this gap by investigating the impact of psychological factors on human longevity. It is an interdisciplinary field that integrates complex psychological, neurological, and molecular relationships that can be best understood with precision medicine methodologies. This perspective argues that psychogenic aging should be considered an integral component of the Hallmarks of Aging framework, opening the doors for future biopsychosocial integration in longevity research. By providing a unique perspective on frequently overlooked aspects of organismal aging, psychogenic aging offers new insights and targets for anti-aging therapeutics on individual and societal levels that can significantly benefit the scientific and medical communities.

## Introduction

The importance of studying aging cannot be underestimated since it is the single most significant risk factor for all-cause mortality and the onset of conditions such as stroke and cancer [1]. The impact of psychological phenomena on healthy longevity has historically been understudied in biomedical science and medicine. But most recently, the consequences of pandemic-related isolation have drawn the attention of many research teams to the connection between mental and physical health [2–4]. Psychological health is now at the forefront of public health discussion as a means to improve human healthspan at the global scale, which presents unique challenges and opportunities.

Epidemiologically, there is robust evidence suggesting that psychological factors disproportionately contribute to mortality and morbidity, yet these associations do not seem to be immutable. For instance, social isolation and overall mental well-being are recognized to be major contributors to all-cause mortality and certain health conditions [5, 6]. Some studies further indicate that psychosocial factors may have a greater effect on mortality risk than other well-established mortality risk factors, such as BMI, physical inactivity, or alcohol consumption [7]. There likewise seems to be a cumulative and dose-dependent effect for these attributions. The Keiser-CDC Adverse Childhood Experience (ACEs) Study was the first study to find a strong relationship between the number of ACE exposures, such as growing up in an abusive household, and a higher incidence of health risk factors and multiple diseases, including cardiometabolic disease [8, 9]. People with 6 or more ACEs have also been found to die 20 years earlier on average compared to those with less traumatic exposure [8]. Additionally, there is molecular evidence linking these early childhood adversities to biological age acceleration and pace of aging acceleration [10]. Importantly, the effects of psychological adversity seem to be modifiable. It has been found, for instance, that individuals who display psychological resilience after early life adversity have no higher odds of developing cardiometabolic disease later in life, compared to those who did not experience adversity [11]. A small, randomized trial found that a multi-modal psychological intervention may modulate the DNA methylome in ways that are relevant to health, aging, and behavior [12]. While these studies cannot definitively establish causality, future randomized trials can focus on the consequence of achieving psychological resilience on biological age markers in humans with more high-throughput methods and larger samples. Taken together, these findings indicate that psychological factors are important and independent contributors to population health and longevity outcomes.

## A Promising Hallmark of Aging

The Hallmarks of Aging is a framework first introduced by López-Otín and colleagues to understand and characterize the biological drivers of aging. These hallmarks include processes to the tune of DNA repair, mitochondria function, telomere attrition, and others. However, as the science evolves, new hallmarks (such as inflammation and microbiome disturbance) have been introduced into this framework [13]. Still, there is skepticism over this paradigm and its practicality [14]. One important limitation is the exclusion of the psychological and behavioral factors that are integral to the aging process. Aging is not only an array of isolated molecular processes—it is an inseparable co-regulation between biological and psychological functions in any given context. Therefore, a reductionist approach to aging that is based solely on its material aspects is inherently inadequate and limiting, particularly in its clinical relevance. This article sees the inclusion of these psychological, social, and behavioral dimensions to the Hallmarks of Aging as a meaningful advancement for the field of aging.

While no agreed-upon criteria exist to define a Hallmark of Aging, there is a general understanding that Hallmarks of Aging should (1) be significantly associated with aging, (2) have biological plausibility, (3) have predictive value, (4) be modifiable, and (5) have broad significance. A person’s total psychological landscape hereby referred to as the *psychome*, seems to fit the benchmarks for serious consideration into the framework.

**Fig. 1:**
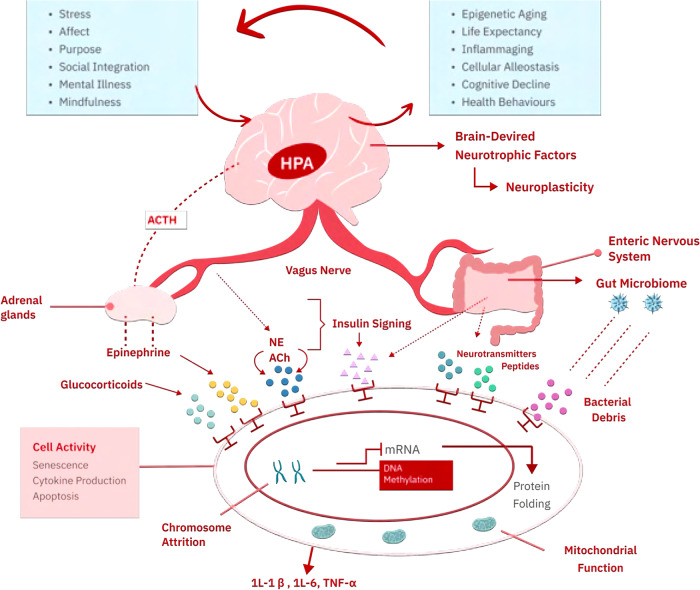
Psychogenic Aging may exert its effects via multiple mechanisms that converge in neuroendocrine dysfunction as regulated by the hypothalamic-pituitary-adrenal (HPA) axis. These psychome disturbances may differentially extend across physiomes and other cellular omes, resulting in different aging phenotypes across level of organization.

First, multiple features of the psychome have been associated with biological age acceleration, including psychological stress [15], depression [16], and anxiety [17]. They are also predictive of lower life expectancy, as is the case with ACEs. In these states, the role of physiological stress is essential, since it is one of the major links between the incidence of age-related diseases, such as dementia and cardiometabolic disease, and mental health [18, 19]. Biologically, these effects may at least be in part moderated by abnormal glucocorticoid signaling [20, 21] and increased inflammatory gene transcription [22, 23] which affects numerous others cellular processes (Fig. 1). The disruption of the normal signaling patterns caused by stress has also been shown to alter the epigenetic landscape to modify the expression of neurotransmitter-regulating genes, which may increase the vulnerability for cognitive decline and other age-related phenotypes [24, 25]. These findings support the epigenetic hypothesis of aging-related cognitive dysfunction and illustrate the profound contribution of psychogenic aging to functional decline [26, 27]. Recent in vivo evidence further suggests that biological age acceleration may be restored upon stress recovery, both in mice and in humans. This finding is consistent with human studies using telomere length and telomerase as aging biomarkers, which likewise find that stress-reducing interventions can have salutatory effects on these surrogate markers [28, 29]. Notably, a randomized controlled trial on stress reduction did find an 18-month cancer survival advantage amongst women in the intervention group, although biological age was not measured in this study [30]. The nexus of all these different lines of evidence points to the existence of a *psychogenic aging* process that fits well within the current Hallmarks of Aging framework. Future studies should focus on the validation of psychogenic aging, elucidate what populations are most genetically and socially vulnerable, and identify which strategies work best to alleviate the physiological impact of psychogenic aging.

Importantly, psychogenic aging is a distinctive and unique hallmark with promising attributes. The existing Hallmarks of Aging are understood as physical changes leading to loss of physiological function [31, 32]. Normative age-related changes in psychological function are also well-defined yet include both loss-of-function and gain-of-function attributes. Put simply, some aspects of psychogenic aging may be considered beneficial, as opposed to all physical hallmarks of aging. For instance, cognitive function may either improve or decline depending on what domain is being examined. Fluid abilities, or the ability to reason and problem-solve, declines with age, whereas crystallized intelligence, or accumulated knowledge, improves over time [33]. This may furthermore interact with meta-cognitions, such as positive age beliefs, which have been linked to a lower risk of dementia in older adults, even in the presence of genetic predisposition [34]. A similar paradigm plays out for meaning, positive affect, and marital satisfaction, whereby all three increase over time and are all independently associated with longevity [35–37]. Mechanistically, this may be related to lower amygdala activity and psychophysiological stress reactivity in older adults [38, 39]. These studies suggest the existence of a pro-longevity mindset that is either an acquired adaptation of long-living individuals, a coping mechanism, or a causal protective factor that may be adopted earlier in life as an anti-aging measure. In light of its unique constituency of both adaptive and mandative age-related changes, psychogenic aging stands out amongst other Hallmarks of Aging as a particularly promising way to increase health and quality of life by capitalizing on innately beneficial aging processes.

## Big Data for Mind and Mortality

**Fig. 2:**
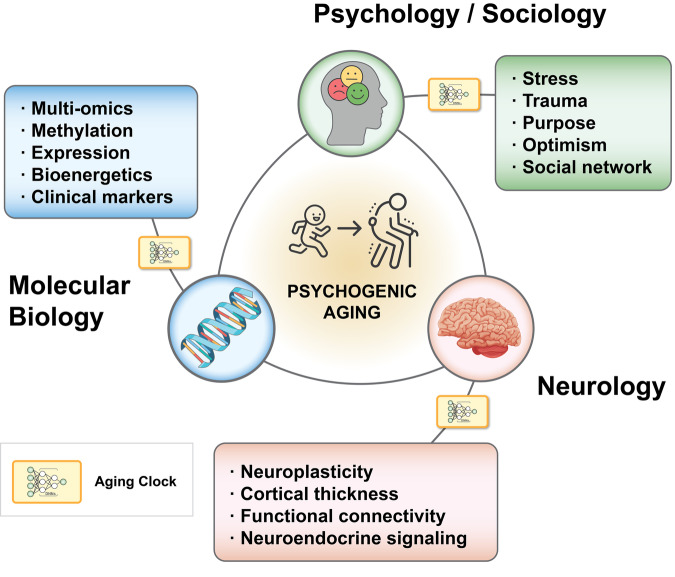
Psychogenic aging involves different levels of biological organization. While each level is studied separately, they all contribute to a single process via feedback loops. Aging clocks are an essential tool that further our understanding of the mechanisms driving the crosstalk between mental health and aging-related degeneration.

While the existing body of work is a promising lever to enhance healthspan, there is still a long way to go before these psychogenic aging dynamics can be understood and implemented with exactitude. This lapse in the literature is an exciting and promising area of inquiry, one that requires an interdisciplinary systems biology approach that integrates psychology, neuroscience, molecular biology, and their relevant subsystems (Fig. 2). This level of complexity is difficult to capture, but this prospect can be most thoroughly understood with precision medicine methodologies, ranging from aging clocks, longitudinal multi-omics, and wearable biobehavioral tracking. Precision approaches, in turn, can be more proactive and predictive than conventional approaches [40] and may reveal complex relations that may be otherwise impossible to untangle.

The most immediate prospect to elucidate these relations is centered around integrating aging clocks and machine learning with psychological, behavioral, and social data. Aging clocks were introduced in biogerontology with a series of seminal papers that described a linear predictor of chronological age trained on epigenetic information [41]. Over a decade of research, aging clocks have received sufficient validation to consider them reliable proxies of a person’s longevity potential. The increased pace of aging reported by aging clocks has been associated with aging-associated conditions, including cardiovascular event risks, metabolic syndrome, and all-cause mortality [42–45]. Both personality traits and mental illness have been linked to biological age clocks [46]. Therefore, aging clock technologies can be employed to reveal connections and relationships between psychological states and aging mechanisms.

For instance, brain transcriptomics have been used to elucidate the relationship between social organization and longer lifespan across different mammalian species [47]. Multiple meta-analyses have found a similar relationship between socialization and longevity in humans, although the transcriptomic mechanisms for this association are yet to be discovered [48]. Aging clocks trained on expression data might prove to be essential in exploring such hypothetical mechanisms [49, 50] and biomark the effect of psychosocial interventions in clinical populations. Other aging clocks may be applied to discover the drivers of psychogenic aging with other omics data types, such as epigenomes, gut metagenomes, and proteomes [51–55]. To further accelerate their translation into clinical practice, these studies should aim to find multi-omic biomarkers for the different discrete mental states that have been implicated in aging and longevity, as part of the greater effort to examine their discriminant validity.

The recent advancements in psychogenic aging research may amount to an improved understanding of the psychobiological contributions to biological age, but also in the creation of aging quantification tools. A study taking such an approach highlighted that mental well-being – including loneliness, sleep, boredom, and happiness measures—has a greater contribution to longevity than previously assumed, in some cases overtaking such strong drivers of aging as smoking [56]. Additionally, other AI clocks based on social and behavioral data have already been developed to predict chronological age and perceptions of age, the latter of which is independently related to longevity [57]. Another advantage of training clocks on psychological data is that such traits predispose people towards certain health or risk behaviors, which may be a more actionable and upstream approach for intervention. While no studies have prospectively examined the relationship between psychogenic aging clocks and human behavior, such efforts should be a priority for future studies, especially given the unresolved problem that is sustainable behavior change—a key challenge for adopting a longevity-promoting lifestyle.

The combination of molecular-level and psychological aging clocks is a frequently discussed approach that might serve as the foundation of a mechanistic psychogenic aging theory [58]. Additionally, the potential of factoring biobehavioral wearable tracking—such as sleep, movement, and stress monitoring—remains a relatively unexploited yet promising avenue to untangle these relationships or create other psychobiological clocks [59]. A multi-modal aging clock that would take into account all possible dimensions of the aging process, however, remains to be developed. The immense potential of such a technology is undeniable since the assessment of future biobehavioral anti-aging interventions is not feasible by any other means.

## Conclusion

Longevity and mental well-being are deeply and inseparably interconnected, as has been shown by an increasingly large number of research teams who are aiming to unveil the mechanisms driving their crosstalk. Yet, to truly integrate these novel insights into the broader practice of geroscience, psychogenic aging should be incorporated into the existing and expanding Hallmarks of Aging framework. Novel advancements in biomedicine, such as aging clocks and high throughput multi-omic designs, have proven to be indispensable in expanding our understanding of the mind’s role in shaping morbidity and mortality, yet much progress remains to be made. The forthcoming waves of psychogenic aging research, as proposed herein, will be crucial for developing therapies targeting mental illness and age-related illnesses, but also ensuring the quality of life and satisfaction of an aging population. The need for more theoretical and translational research in this novel interdisciplinary field is timely and apparent, calling for new advances to patient care that address the concurrent aging and mental illness trends across the globe.
